# ISCB Honors Michael S. Waterman and Mathieu Blanchette

**DOI:** 10.1371/journal.pcbi.0020105

**Published:** 2006-08-04

**Authors:** Merry Maisel

Each year, the International Society for Computational Biology (ISCB) takes nominations for its two major awards. An awards committee, composed of a group of current and past directors of the Society along with previous recipients, evaluates the nominations and selects the winners. In 2006, the awards committee honors two outstanding scientists.

## Senior Scientist Accomplishment Award: Michael S. Waterman

**Michael S. Waterman:**
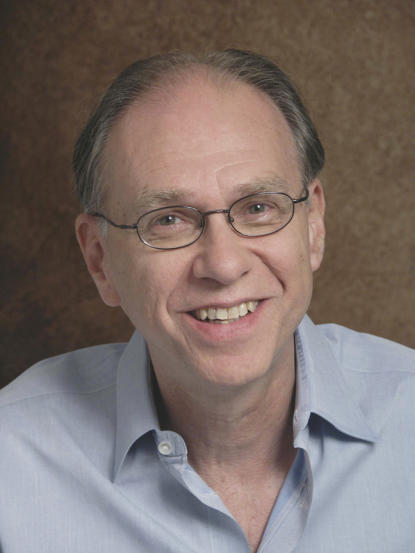


The founding editor of the *Journal of Computational Biology* and one of the originators of the discipline, Professor Michael S. Waterman, has been named winner of the annual Senior Scientist Accomplishment Award. Waterman is professor of biological sciences, computer science, and mathematics at the University of Southern California.

“Professor Waterman has contributed work of prime importance in half a dozen fields of computational biology,” said Thomas Lengauer, professor of computational biology and applied algorithmics at the Max-Planck-Institut für Informatik and chair of the ISCB Awards Committee. “So much of our work is based on methods for finding sequence homology that, if we weren't constantly citing it, it would amaze us that the methodology was first devised only 25 years ago by Mike Waterman and Temple Smith. Since then, Waterman developed the dynamic programming approach to RNA structure prediction, began the combinatorial study of RNA secondary structure, improved the statistical tests incorporated into BLAST and related tools, and worked on the assembly problem for genomes past and present. He has also made important contributions to phylogeny, tree comparison, motif searching, cryptogene analysis, parametric alignment, gapped alignment, optical mapping, haplotype estimation, gene family evolution, and a host of other problems whose solutions have brought our discipline immense power and respect.”

The ISCB award recognizes members of the computational biology community who have made major contributions to the field through research, education, service, or a combination of the three.

“Michael Waterman's contribution to the field goes well beyond being a researcher, educator, and journal editor,” said Pavel Pevzner, who is Ronald R. Taylor Professor of Computer Science at the University of California San Diego.

“He and David Sankoff are responsible for transforming bioinformatics from a ‘stamp collection' of ill-defined problems into a rigorous discipline with important biological applications. Without such a transformation,” Pevzner said, “bioinformatics would never be able to attract the top talent in computer science and statistics or the other members of the generation of talented young scientists who are working in the field today.”

Waterman obtained a bachelor's in mathematics from Oregon State University and a doctorate in statistics and probability from Michigan State University, then began his academic career at Idaho State University. He was invited to spend several summers at Los Alamos National Laboratory in the early 1970s. In a memoir published on his Web site, Waterman has written: “I was an innocent mathematician until the summer of 1974. It was then that I met Temple Ferris Smith and for two months was cooped up with him in an office at Los Alamos. . . . that experience transformed my research, my life, and perhaps my sanity.”

Smith, now director of the BioMolecular Engineering Research Center at Boston University, was also visiting Los Alamos from a small university in Michigan.

At Los Alamos, their fellow scientists and friends included Stanslaw Ulam, Nick Metropolis, Marc Kac, and Gian-Carlo Rota, all towering names in computational science, at a time when the lab was a hotbed of intellectual ferment. Waterman relates that it was Smith who, although trained in nuclear physics, introduced the group to the prospects of applying mathematics to biological questions. One result was the Smith–Waterman algorithm for determining the degree of similarity (homology) of amino acid sequences from DNA, RNA, or proteins. In their justly famous three-page paper, published in the *Journal of Molecular Biology* in 1981, Waterman and Smith changed the face of molecular biology and participated in launching the bioinformatics revolution.

Waterman joined the staff at Los Alamos in 1975, then moved to the University of Southern California in 1982. He has been honored as a USC Professor and holds the USC Associates Endowed Chair in the Natural Sciences. In 2003, Professor Waterman became Faculty Master of Parkside International Residence College at USC, which is home to 600 students and an international center.

“Computational biologists today are all beneficiaries of his work,” Lengauer said, “He has trained more than a handful of prominent computational genomicists, served on virtually all the panels and committees that guide government in evaluating grants and fellowships, and has generally guided the development of the discipline. He has been an active member of ISCB since its founding, and he worked with Pavel Pevzner and Sorin Istrail to start RECOMB, the Conference on Research in Computational Molecular Biology, which held its tenth meeting in April.”

Waterman said: “It is an honor to join the select company of the previous award winners: David Sankoff, David Lipman, and Janet Thornton. We have all been fortunate in our choice of the right problems.”

The award will be presented to Waterman at the ISCB's annual meeting, Intelligent Systems for Molecular Biology (ISMB) in Fortaleza, Brazil, from August 6 through 10, 2006. Dr. Waterman will deliver the annual Senior Scientist Accomplishment Award keynote lecture, titled Whole Genome Optimal Mapping, as the finale to the conference.

## Overton Award: Mathieu Blanchette

**Mathieu Blanchette:**
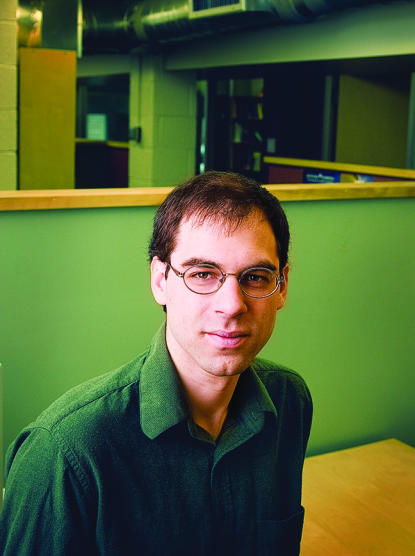


Mathieu Blanchette, assistant professor in the School of Computer Science at McGill University in Montréal, will receive the 2006 Overton Prize.

“Mathieu Blanchette is responsible for fundamental, highly cited contributions in several areas of bioinformatics,” said Professor Thomas Lengauer of the Max-Planck-Institut für Informatik, who is chair of the ISCB Awards Committee.

“His doctoral thesis contained perhaps the first reasonable algorithm for gene order phylogeny, based on a solution to the breakpoint median problem, and it also elaborated the now-famous concept of phylogenetic footprinting. As a postdoctoral researcher, he played a central role in working out algorithms for reconstructing ancestral mammalian genomes. His most recent work continues his interest in the inference of evolutionary scenarios and gene regulation. And he has been active in the bioinformatics community since his student days, presenting papers at the Computing and Combinatorics conferences and the Research in Computational Molecular Biology meetings, among others. He is currently organizing several workshops and conferences, has attracted many students to his new lab, and has been highly successful in obtaining funding in a competitive environment,” Lengauer said.

The Overton Prize was established in 2001 by the ISCB in memory of G. Christian Overton, who was Director of the Center for Bioinformatics at the University of Pennsylvania and a major contributor to the field. He was a member of the ISCB Board of Directors, and his sudden death in 2000 was a shock to the community. The prize is awarded annually to a scientist in the early to middle stage of his or her career who has contributed significantly to computational biology through research, education, service, or a combination of the three.

The prize will be awarded at the ISCB annual meeting, Intelligent Systems for Molecular Biology, to be held in Fortaleza, Brazil, from August 6 through 10, 2006. Blanchette will give the Overton keynote lecture, titled What Mammalian Genomes Tell Us about Our Ancestors and Vice Versa, on August 8.

From 1994 through 1997, Blanchette was an undergraduate in the Mathematics and Computer Science departments of the Université de Montréal. After graduating, he did an M.Sc. there as well, writing a thesis on breakpoint phylogeny under the direction of David Sankoff. He then went to the University of Washington, obtaining a Ph.D. in Computer Science (2002) under the supervision of Martin Tompa. He spent the next year as a postdoctoral researcher at the Center for Biomolecular Science and Engineering of the University of California Santa Cruz, where he worked with David Haussler. He took up his current position at McGill in 2003.

Blanchette said: “Computational biology is, I believe, the greatest topic a computer scientist can study. It has the hardest algorithmic challenges and the most important implications, and it brings together the most fun collaborations. The main challenge, though, is asking the right question: one that is biologically relevant, mathematically clear, and algorithmically solvable.”

Regarding the award, he noted: “Chris Overton was one of the first explorers of the world of bioinformatics, before the name even existed, and he opened the area to young people like me. I am immensely grateful for the work he did and greatly honored to receive this award in his memory. I owe this, and everything I am, to my invaluable interactions with three key mentors, who still have much to teach me. David Sankoff knows how to ask the right questions. Martin Tompa can solve them the right way. And David Haussler has the most insightful appreciation for the biological implications of our work.”

Blanchette's group is interested in understanding how genomes evolved, to better understand how they work. “Evolution is giving us a huge number of clues about how our genome functions,” he said. “We are only starting to take advantage of these clues. The computational reconstruction of ancestral mammalian genomes will help tremendously in the functional annotation of the human genome. The goal of this work, which was begun with David Haussler and Webb Miller of Pennsylvania State University, is to develop the computational, algorithmic, and statistical tools we need to take full advantage of the wealth of information older genomes contain.”

He pointed out: “Genomes don't just contain protein-coding genes! Surprises regarding the various functions encoded in our genome keep coming at an unrelenting pace, indicating that we have a long way to go to fully understanding it—but the impacts of the genome's ‘user manual,' of which only the first chapters have been written, will be felt in all of science and medicine.

## Abbreviations

ISCB: International Society for Computational Biology

